# GWAS analyses reveal QTL in egg layers that differ in response to diet differences

**DOI:** 10.1186/s12711-015-0160-2

**Published:** 2015-10-19

**Authors:** Hélène Romé, Amandine Varenne, Frédéric Hérault, Hervé Chapuis, Christophe Alleno, Patrice Dehais, Alain Vignal, Thierry Burlot, Pascale Le Roy

**Affiliations:** INRA, UMR1348 PEGASE, Domaine de La Prise, 35590 Saint-Gilles, France; Agrocampus Ouest, UMR1348 PEGASE, 65 Rue de Saint Brieuc, 35042 Rennes, France; Novogen, Mauguérand, 22800 Le Foeil, France; SYSAAF, INRA UR83 Recherches Avicoles, 37380 Nouzilly, France; Zootests, Parc Technologique Du Zoopôle, 5 Rue Gabriel Calloet Kerbrat, 22440 Ploufragan, France; INRA, UMR1388 GenPhySe, Auzeville BP52627, 31326 Castanet-Tolosan, France

## Abstract

**Background:**

The genetic architecture of egg production and egg quality traits, i.e. the quantitative trait loci (QTL) that influence these traits, is still poorly known. To date, 33 studies have focused on the detection of QTL for laying traits in chickens, but less than 10 genes have been identified. The availability of a high-density SNP (single nucleotide polymorphism) chicken array developed by Affymetrix, i.e. the 600K Affymetrix^®^ Axiom^®^ HD genotyping array offers the possibility to narrow down the localization of previously detected QTL and to detect new QTL. This high-density array is also anticipated to take research beyond the classical hypothesis of additivity of QTL effects or of QTL and environmental effects. The aim of our study was to search for QTL that influence laying traits using the 600K SNP chip and to investigate whether the effects of these QTL differed between diets and age at egg collection.

**Results:**

One hundred and thirty-one QTL were detected for 16 laying traits and were spread across all marked chromosomes, except chromosomes 16 and 25. The percentage of variance explained by a QTL varied from 2 to 10 % for the various traits, depending on diet and age at egg collection. Chromosomes 3, 9, 10 and Z were overrepresented, with more than eight QTL on each one. Among the 131 QTL, 60 had a significantly different effect, depending on diet or age at egg collection. For egg production traits, when the QTL × environment interaction was significant, numerous inversions of sign of the SNP effects were observed, whereas for egg quality traits, the QTL × environment interaction was mostly due to a difference of magnitude of the SNP effects.

**Conclusions:**

Our results show that numerous QTL influence egg production and egg quality traits and that the genomic regions, which are involved in shaping the ability of layer chickens to adapt to their environment for egg production, vary depending on the environmental conditions. The next question will be to address what the impact of these genotype × environment interactions is on selection.

**Electronic supplementary material:**

The online version of this article (doi:10.1186/s12711-015-0160-2) contains supplementary material, which is available to authorized users.

## Background

Over the last decades, layer chicken lines have been selected and improved for egg production and egg quality performance. However, the genetic architectures of the underlying traits, i.e. the quantitative trait loci (QTL) that influence these traits, are still poorly known. To date, 33 studies have focused on the detection of QTL for laying traits in chickens, but less than 10 genes have been identified [1].

The high-density array for chicken recently developed by Affymetrix, i.e. the 600K Affymetrix^®^ Axiom^®^ HD genotyping array [2], offers the possibility to use high-density genotype data for genomic selection in laying hens. It will also contribute to improve the localization of previously detected QTL and to detect new QTL. This high-density array is also anticipated to take research beyond the classical hypothesis of additivity of QTL effects or of QTL and environmental effects. Indeed, some studies suggest that genotype × environment (G × E) interactions may explain a large part of the phenotypic variance in laying traits in chickens [3]. However, to date, no study has tested the robustness of QTL across environments.

Therefore, in this study, a genome-wide association study (GWAS) using the 600K Affymetrix^®^ Axiom^®^ HD genotyping array was conducted to detect QTL that influence egg production and egg quality traits in layer chickens. In order to investigate whether the QTL detected differed between environmental conditions, animals were divided into two groups that were fed a different diet.

## Methods

### Animals

The population studied consisted of 438 sires from a commercial pure line that was created and selected by NOVOGEN (Le Foeil, France) and 31,381 of their F1 crossbred female offspring. Hens were hatched in three batches in November 2010, May 2011 and November 2011. At 18 weeks of age, they were housed in a production farm in collective cages that contained 12 half-sisters of the same sire. The hens laid from 18 to 75 weeks of age. Fifty percent of the hens were fed ad libitum a high-energy diet (HE) that supplied 2881 kcal of metabolizable energy (ME) (1342 cages) and 50 % were fed ad libitum a low-energy diet (LE) that supplied 2455 kcal of ME (1346 cages).

### Genotyping

Blood was sampled from the brachial veins of the sires, DNA was extracted and hybridized on the 600K Affymetrix^®^ Axiom^®^ HD genotyping array [2] by Ark-Genomics (Edinburgh, UK). In total, 438 sires were genotyped for 580,961 SNPs that were distributed over chromosomes 1 to 28, two linkage groups (LGE22C19W28_E50C23 and LGE64) and the two sex chromosomes, along with a group of 7883 markers of unknown location. Genotypes were filtered in five successive steps: (1) 14 SNPs on chromosome W with a call rate less than 5 % were excluded; (2) none of the animals had a call rate less than 95 %; (3) 260,945 SNPs with a minor allele frequency less than 0.05 were excluded; (4) 9041 SNPs with a call rate less than 95 % were excluded; and (5) 26,318 SNPs that deviated significantly (P < 5 %) from Hardy–Weinberg equilibrium were excluded. Finally, 284,643 SNPs remained for analysis and no individual was excluded.

### Measurement of traits

In this paper, traits are named according to Animal Trait Ontology for Livestock [4].

Egg production was recorded daily from week 18 to week 75 and egg production rate (EPR) of one cage (in  %) was calculated by dividing the number of eggs produced by the number of hen days in the cage. Based on the laying curve, four periods of production were defined: increasing from week 18 to week 30 (EPR1); plateau from week 31 to week 49 (EPR2); persistence from week 50 to week 75 (EPR3); and global production from week 18 to week 75 (EPR).

At about 50 weeks of age, and then again at 70 weeks, all eggs produced on the farm were collected and egg quality traits were measured by the Zootests company (Ploufragan, France) on 27,747 week-50 eggs and 25,964 week-70 eggs (Table 1). The first step consisted of measuring the short length of the egg (SLE, in mm) and egg weight (EW, in g), before calculating egg shell shape (ESshape) as: ESshape = (SLE/10)/(EW/10)^1/3^. Second, shell color was measured with a Minolta chromameter and three traits were recorded: redness of egg shell a* (RSS), yellowness of egg shell b* (YSS) and lightness of egg shell L* (LSS). Egg shell color was then calculated as: ${\text{ESC}} = 100 - \left( {{\text{L*}} - {\text{A*}} - {\text{B*}}} \right)$. Third, shell strength was measured using a compression machine to evaluate the static stiffness of the shell. The egg was compressed between two flat plates moving at constant speed and at a constant force of 15 N to record egg shell stiffness (ESSTIF, in mm). Egg shell strength is the maximum force recorded before fracture of the shell (ESS in N). Then, each egg was broken and albumen height (H) was measured using a tripod. The Haugh unit (HU) measure of albumen firmness was then calculated as: HU = 100 log (H − 1.7 EW^0.37^ + 7.57) [5]. The yolk was weighed and a yolk index (YOLKIND) was calculated as yolk weight divided by EW. Finally, the egg was scored for blood and meat spots (EMTSP) on a scale of 0 (without spots)–3 (many spots).

**Table 1:** Summary statistics on phenotypic data for the high energy (HE) and low energy (LE) diets

Trait	Number of records^a^		Mean		Standard deviation	
Diet	HE	LE	HE	LE	HE	LE
Egg production traits						
EPR (%)	1342	1340	84.22	83.26	6.12	7.17
EPR1 (%)	1344	1342	72.12	76.44	9.40	9.35
EPR2 (%)	1342	1338	91.14	89.28	6.06	8.16
EPR3 (%)	1344	1342	85.32	81.97	6.35	7.62
Egg quality traits at 50 weeks of age						
ESshape	12,375	12,403	1.1	1.1	0.02	0.02
EW (g)	13,547	13,603	61.11	60.59	4.78	4.74
SLE (mm)	12,378	12,409	43.37	43.32	1.29	1.28
ESC	13,528	13,580	24.78	25.31	9.66	10.09
LSS	13,548	13,596	66.77	66.91	4.57	4.72
RSS	13,546	13,605	13.72	13.42	3.16	3.27
YSS	13,445	13,493	28.3	28.22	2.65	2.81
ESS (N)	12,379	12,410	39.15	39.58	7.51	7.33
ESSTIF (mm)	12,388	12,418	193.42	191.49	29.53	28.57
EMTSP	6959	6283	1.68	1.55	0.74	0.72
HU	6938	6257	73.03	74.45	7.69	8.02
YOLKIND	6157	6808	0.27	0.27	0.02	0.02
Egg quality traits at 70 weeks of age						
ESshape	11,315	10,983	1.1	1.1	0.02	0.02
EW (g)	12,945	12,804	61.05	60.61	5.04	4.95
SLE (mm)	11,329	11,004	43.41	43.36	1.37	1.35
ESC	12,929	12,259	25.4	25.48	10.18	10.64
LSS	12,937	12,272	67.09	66.99	4.92	5.05
RSS	12,942	12,274	13.4	13.3	3.27	3.42
YSS	12,833	12,188	28.35	28.23	2.65	2.86
ESS (N)	11,327	11,007	37.4	37.82	7.53	7.75
ESSTIF (mm)	11,332	11,004	192.83	190.46	29.98	30.53
EMTSP	6459	6705	1.63	1.63	0.72	0.71
HU	6437	6683	64.88	67.28	9.31	9.46
YOLKIND	5729	5491	0.27	0.27	0.02	0.02

^a^Number of cages for production traits and number of eggs for quality traits

### Statistical analysis

First, egg measurements were adjusted for environmental effects. Separately for each hatch, the covariates and fixed effects were tested using the SAS^®^ 9.2 GLM procedure based on the following linear models: ${\text{Y}}_{\text{ijlmn}} = {\text{ s}}_{\text{i}} + {\text{ d}}_{\text{j}} + {\text{ b}}_{\text{l}} + {\text{ c}}_{\text{m}} + {\text{ f}}_{\text{n}} + {\text{ E}}_{\text{ijlmn}}$, for EPR, EPR1, EPR2 and EPR3 and ${\text{Y}}_{\text{ijklmno}} = {\text{ s}}_{\text{i}} + {\text{ d}}_{\text{j}} + {\text{ a}}_{\text{k}} + {\text{ b}}_{\text{l}} + {\text{ c}}_{\text{m}} + {\text{ f}}_{\text{n}} + {\text{ e}}_{\text{o}} + {\upbeta}_{ 1} {\text{W}}_{\text{ijklmno}} + {\upbeta}_{ 2} {\text{R}}_{\text{ijklmno}} + {\text{ E}}_{\text{ijklmno}}$, for SLE, EW, ESshape, RSS, YSS, LSS, ESC, ESSTIF, ESS, HU, YOLKIND and EMTSP, where Y_ijlmn_ and Y_ijklmno_ are trait values, s_i_ is the fixed effect of sire i (438 levels); d_j_ is the fixed effect of diet j (two levels: HE or LE); a_k_ is the fixed effect of age class k (two levels: 50 or 70 weeks); b_l_, c_m_ and f_n_ represent the location of the cage in the building, respectively the fixed effect of battery l (four levels), the fixed effect of column m (two levels: middle or edges of the battery) and the fixed effect of floor n (two levels); e_o_ is the fixed effect of the person who made the measurement (eight to 10 levels according to quality trait), W_ijklmno_ and R_ijklmno_ are the waiting time between sample and egg measurement (in days) covariate and the age of the hen (in days) covariate; E_ijlmn_ and E_ijklmno_ are random residual variables.

For each trait, a sub-model that took only effects exceeding the significance level (P < 0.2) into account was retained. Raw data were then adjusted using the estimates of all effects in this model, except the sire effect. Distributions of the adjusted data were tested for each trait and extreme individual values, i.e. values that were more than four phenotypic standard deviations from the mean, were discarded. Finally, for each trait, the “performance” of one sire was calculated as the mean of its daughters’ adjusted performances.

To examine the genetic architecture of a trait, all data were considered together, i.e. one mean per sire across the two diets and ages, whereas to examine the QTL effect for each condition, data were considered separately for each condition, i.e. two means per sire (HE and LE diets) for egg production traits and four means per sire (for egg collection at 50 and 70 weeks of age, and HE and LE diets) for egg quality traits.

### GWAS analysis

According to the recommendations made by Teyssedre et al. [6], data were analyzed using a mixed model that takes pedigree kinship into account. For each SNP (1 to 284,643) and each trait (1 to 16), the following mixed model was applied: ${\mathbf{Z}} = {\mathbf{1}}{\upmu}_{{}} + {\mathbf{X}}{{\upalpha }} + {\mathbf{E}}_{{}}$, with ${\text{V}}\left( {\mathbf{E}} \right) \, = {\mathbf{A}}{{\upsigma }}^{ 2}_{\text{g}} + {\mathbf{I}}{{\upsigma }}^{ 2}_{\text{e}}$, where **Z** is the vector of the sire performances; µ is the general mean; **X** is the incidence matrix of genotypes for the SNP evaluated; α is the allele substitution effect of the SNP for the trait [7]; **E** is the vector of residuals with variance–covariance matrix V(**E**); **A** is the pedigree kinship matrix; **I** is the identity matrix; ${\upsigma}_{\text{g}}^{2}$ is the genetic variance and ${\upsigma}_{\text{e}}^{2}$ is the environmental variance.

This model was fitted using the BLUPF90 program [8]. Chromosome-wide thresholds (P < 1 %) to test the H0 hypothesis, i.e. no effect of the SNP on the trait, were estimated using the method of Müller et al. [9] and Müller’s software [10], which offsets the heavy computations to parallel implementation. Genome-wide thresholds (P < 5 %) were calculated according to the Bonferroni correction as chromosome-wide thresholds at P < 0.0015 (0.05/32 chromosomes). For the group of SNPs with unknown locations, the threshold was set at P < 1e–5 (0.05/3681 SNPs), according to the Bonferroni correction.

### QTL detection

For each chromosome, a QTL was detected when one SNP was genome-wide significant (P < 5 %). The confidence interval of this QTL was defined with the adjacent SNPs that were chromosome-wide significant (P < 1 %). For each QTL, the SNP with the highest estimated effect (α) was called the “top SNP”. Based on the estimate of the allele substitution effect for the top SNP, α, the genetic variance explained by the top SNP was calculated as 2p(1 − p)α^2^, where p is the minor allele frequency. The percentage of variance explained by the top SNP was then calculated for each trait by dividing by the variance of the sire performances, and called “% of explained variance”.

### QTL × environment interaction

For each trait, the GWAS analysis was repeated separately, as above, for the four datasets for each condition (egg collections at 50 and 70 weeks of age, and HE and LE diets) in order to estimate the effect of the top SNP for each QTL in the two environments, α_1_ and α_2_, i.e. egg collection at 50 *vs.* 70 weeks of age and HE *vs.* LE diet. QTL × age and QTL × diet interactions were tested by comparing the two corresponding estimates using a Z test statistic. Variances explained by the top SNPs for each condition were calculated, and the residual variances for the different conditions were compared with a Fisher test to verify their equality (which was always accepted). The Z test statistic was calculated as follows:

$${\text{Z}} = \frac{{ | {\upalpha}_{ 1} -{{\upalpha }}_{ 2} |}}{{\upsigma}} \times\sqrt {\text{n}} ,$$

where σ^2^ is the average residual variance and n is the average number of sires over the two conditions. Z followed a normal distribution because n was large. However, if the interaction effect is large, some QTL may not be detected by analyzing the whole dataset. Indeed, additional QTL, i.e. QTL that were not detected when analyzing the whole dataset, were identified by analyzing the four within-condition datasets separately. In such cases, we also tested the QTL × environment interaction. The QTL were defined using the results of the within-condition GWAS analyses, and the estimated effects of the top SNP for each QTL were compared using the Z test statistic described previously.

## Results and discussion

Number of observations, means and standard deviations of traits are in Table 1 for the raw data. Differences in average performance between the HE and LE diets ranged from 0 to 0.5 standard deviations. Differences in performance between the two age groups (egg collections at 50 and 70 weeks of age) ranged from 0 to 0.9 standard deviations. Overall, these differences remained small, even if, in general, they were significant.

Correlations between the sires’ “performances” depending on diet and age at collection are in Table 2. They are much lower for production traits than for egg quality traits, except for EMTSP. For quality traits, they are lower for alternate diets than for alternate ages and they vary more between traits. At a broad level, these correlations confirm the putative presence of interaction effects.

**Table 2:** Phenotypic correlations between the “performances” of sires for alternate diets and alternate ages

	Diet^a^	Age^b^
Egg production traits		
EPR	0.42	–
EPR1	0.25	–
EPR2	0.28	–
EPR3	0.10	–
Egg quality traits		
ESshape	0.67	0.78
EW	0.78	0.92
SLE	0.76	0.87
ESC	0.73	0.83
LSS	0.74	0.84
RSS	0.72	0.82
YSS	0.56	0.62
ESS	0.67	0.77
ESSTIF	0.71	0.77
EMTSP	0.42	0.47
HU	0.50	0.57
YOLKIND	0.56	0.67

^a^Correlations between the sires’ performances depending on the diet

^b^Correlations between the sires’ performances depending on the age of measurement

### GWAS analyses

For egg production traits, the GWAS analysis on the whole dataset resulted in 1202 significant tests at the 1 % chromosome significance level and identified 861 SNPs with an effect on at least one egg production trait. For egg quality traits, it resulted in 8116 significant tests at the 1 % chromosome significance level and identified 5384 SNPs with an effect on at least one trait.

**Fig. 1:**
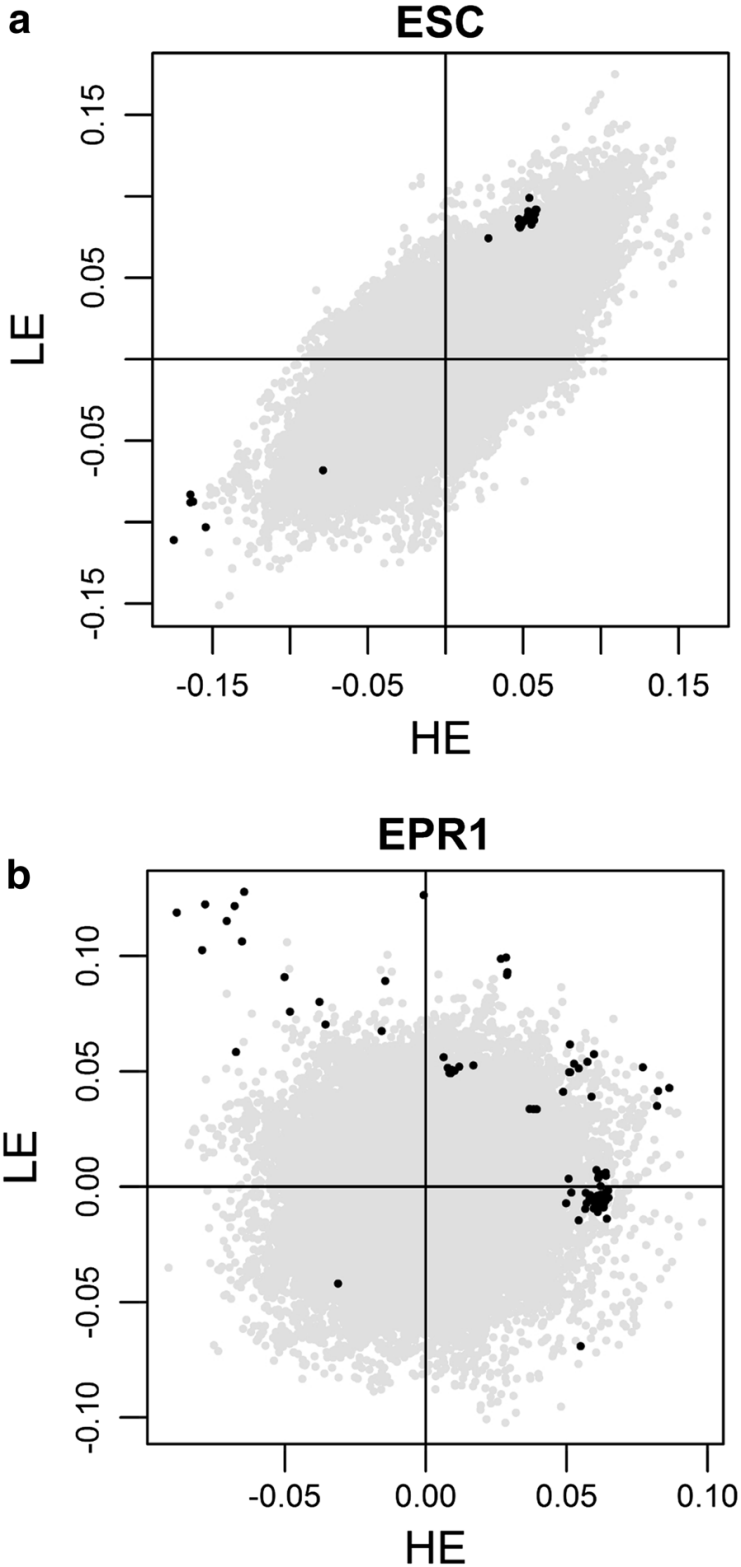
Examples of correlations between estimates of SNP effects for egg shell color (**a**) and egg production rate 1 (**b**) for the high-energy diet (*x*-axis) and the low-energy diet (*y*-axis). Chromosome-wide significant SNPs (P < 1 %) located in QTL that show an interaction with diet are plotted in *black*; correlations between estimates of SNPs effects are equal to 0.75 for egg shell color and to 0.16 for egg production rate

Analyses of the four subsets estimated the effect of each SNP for each of the four conditions investigated (HE diet, LE diet, egg collections at 50 and 70 weeks of age). Correlations between estimates of SNP effects based on HE and LE diets ranged from 0.16 to 0.80 (Table 3). They were lower for egg production traits than for egg quality traits. Figure 1 shows an example of the correlations between estimates of SNP effect for the HE and LE diets for egg shell color and EPR1. Estimates of the effects of each SNP for each diet were plotted and SNPs within a QTL that had an interaction with diet were investigated, in order to determine the direction of the interaction. For egg quality traits, in most cases, the sign of the allele substitution effect was not reversed between diets. Instead, interactions were due to deviations of the magnitude of the SNP effects, except for two traits with a low correlation between diets, YOLKIND and EMTSP. However, for egg production traits, in most cases, the sign of the allele substitution effect was reversed. Correlations between estimates of the SNP effects at 50 and 70 weeks of age ranged from 0.54 to 0.93.

**Table 3:** Correlations between the effects of SNPs for alternate diets and alternate ages

	Diet^a^	Age^b^
Egg production traits		
EPR	0.35	–
EPR1	0.16	–
EPR2	0.27	–
EPR3	0.27	–
Egg quality traits		
ESshape	0.69	0.8
EW	0.8	0.93
SLE	0.78	0.88
ESC	0.75	0.86
LSS	0.8	0.87
RSS	0.76	0.86
YSS	0.61	0.74
ESS	0.66	0.78
ESSTIF	0.7	0.76
EMTSP	0.46	0.54
HU	0.54	0.62
YOLKIND	0.6	0.72

^a^Correlations between the effects of SNPs depending on the diet

^b^Correlations between the effects of SNPs depending on the age of measurement

### QTL detected

To analyze the genetic architecture of each trait, all the data were analyzed together, whereas to determine which QTL were specific to performances for egg collection at 50 and 70 weeks of age or with HE and LE diets, their detection was carried out by using the data divided into four subsets. To calculate the number of QTL detected and to give them a name, we considered the QTL according to their chromosomal localization, regardless of which traits they influenced. For QTL that were identified by analyzing the whole dataset, 11 had a significant effect on at least one egg production trait and 58 QTL had an effect on at least one egg quality trait (see Additional file 1). When GWAS was applied separately to the four subsets, 19 additional QTL with a significant effect on at least one egg production trait and 46 additional QTL with an effect on at least one egg quality trait were detected (see Additional files 2, 3). Three QTL were common to egg production traits and egg quality traits.

QTL localizations are in Additional files 1, 2 and 3, along with estimates of the variance explained by each QTL for each trait for each condition (HE diet, LE diet, egg collections at 50 and 70 weeks of age) are in Table 4.

**Table 4:** QTL detected for each trait: number of QTL detected, number of QTL that show an interaction, percentage of the variance explained across conditions and percentage of the variance explained within each condition

Trait	Nb QTL	QTL diet	QTL age	Var (%)	Var LE (%)	Var HE (%)	Var 50 (%)	Var 70 (%)
Egg production traits								
EPR	9	7	–	26.8	34.07	14.61	NA	NA
EPR1	23	19	–	33.99	85.31	44.61	NA	NA
EPR2	6	3	–	24.07	22.03	10.57	NA	NA
EPR3	7	7	–	16.25	34.04	4.97	NA	NA
Egg quality traits								
ESshape	15	3	1	65.36	45.19	64.42	59.55	59.21
EW	12	1	0	55	49.5	50.58	58.48	47.96
SLE	7	0	0	28.7	26.37	24.23	29.05	25.2
ESC	27	3	1	105.35	96	91.37	93.76	101.89
LSS	24	0	0	102.11	90.47	90.02	94.44	95.84
RSS	19	3	1	85.97	81.79	68.54	76.94	81.68
YSS	15	6	0	61.1	58.58	43.54	51.71	51.02
ESS	8	3	3	27.16	22.7	26.37	21.88	27.47
ESSTIF	8	2	1	32.88	33.82	23.73	29.36	30.16
EMTSP	9	3	2	42.55	24.87	31.16	33.93	27.09
HU	11	2	7	39.26	24.8	34.79	36.08	28.62
YOLKIND	16	2	2	62.45	72.49	40.71	56.95	47.64

*Nb QTL* number of QTL detected for each trait, *QTL diet* number of QTL that show an interaction with diet; *QTL age* number of QTL that show an interaction with age, *var LE* variance explained for the low energy diet (%), *var HE* variance explained for the high energy diet, in percent; *var 50* variance explained at 50 weeks of age (%), *var 70* variance explained at 70 weeks of age (%)

#### Egg production (EPR, EPR1, EPR2, EPR3) traits

For egg production traits, analysis of the whole dataset identified 11 QTL that had an effect on at least one trait and separate analysis of the four subsets detected 21 additional QTL. Previously, several QTL for egg production have been reported [11, 12] that are located close to QTL65 and QTL67 identified here.

For EPR1, 23 QTL were detected on 15 chromosomes, which indicates that it has a polygenic determinism. Together, these QTL explained 34 % of the variance of EPR1, regardless of environmental condition. For EPR and EPR2, nine and six QTL were detected, which explained 26.8 and 24.1 % of the variance, respectively. Although fewer QTL were detected for EPR and EPR2, they had a larger effect when the data are considered together. QTL7 had an effect on the four traits (EPR, EPR1, EPR2 and EPR3) but its effect was diet-dependent. Generally, QTL influencing EPR3 also had an effect on EPR1.

#### Egg weight (EW) and egg form (SLE and ESshape) traits

Analysis of the whole dataset identified 13 QTL that had an effect on at least one of the following traits EW, SLE and ESshape and analysis of the four subsets separately detected 14 additional QTL. Together, these QTL explained 65.4 % of the variance of ESshape, 55 % of the variance of EW but only 28.7 % of the variance of SLE.

QTL106 was common to all three egg weight and shape traits and was located on chromosome 26, between 1.2 and 1.3 Mb. Two QTL for EW have been reported on chromosome 26 at 2 and 3 Mb [13]. QTL 106 explained about 4 % of the variance of EW and SLE, but only 1.6 % of the variance of ESshape.

Three other QTL common to EW and SLE were detected (see Additional file 1), of which two, QTL121 and QTL122, were located on chromosome Z. This agrees with studies that have shown that chromosome Z plays a role in the regulation of EW [14, 15].

#### Egg shell color (ESC, RSS, LSS and YSS) traits

For egg shell color traits, analysis of the whole dataset identified 22 QTL that had an effect on at least one trait and analysis of the four subsets separately detected 17 additional QTL. Several QTL have been reported for egg shell color [16, 17] that are localized close to QTL73 and QTL10. Together, these QTL explained more than 100 % of the variance of ESC and LSS, which, although their effects may be overestimated, suggests the existence of other interactions such as dominance or epistasis, which were not considered in the model.

The number of QTL detected and their distribution along the genome suggest that egg shell color has a polygenic determinism. Most of the QTL for ESC also had an effect on RSS and YSS or on YSS, but only a few had effects on RSS, LSS and YSS. Indeed, only two QTL (QTL20 and QTL119) had an effect on all four traits.

#### Egg shell solidity (ESSTIF and ESS) traits

For egg shell solidity traits, analysis of the whole dataset identified seven QTL that had an effect on at least one trait, i.e. five for ESSTIF and two for ESS but no QTL was common to both traits. Analysis of the four subsets separately detected seven additional QTL that had a significant effect on at least one of these traits, i.e. two for ESSTIF and five for ESS. Overall, the same number of QTL was found for ESS and ESSTIF, but the total variance explained by these QTL was larger for ESSTIF than for ESS. QTL103 was common to both traits and had a pleiotropic effect.

#### Internal egg quality (YOLKIND, HU and EMTSP) traits

For internal egg quality traits, analysis of the whole dataset identified 18 QTL and analysis of the four subsets separately detected 15 additional QTL that had a significant effect on YOLKIND and/or HU. One of these QTL (QTL123) was previously reported by [18]. YOLKIND, for which 16 QTL were identified, is the trait that had the largest proportion of variance explained, followed by EMTSP for which nine QTL were detected.

Among the 33 QTL detected for the three internal egg quality traits, QTL33 was common to HU and YOLKIND and QTL100 was common to HU and EMTSP. For YOLKIND, QTL88 and QTL89 were localized on chromosome 20 between 11 and 12 Mb, nearby a QTL detected for EMTSP. Previously, a QTL for HU, on chromosome 20 at 11 Mb was reported by [13]. Together, these results suggest that this region has a pleiotropic effect.

### QTL × environment interactions

Our main aim was to determine whether diet and age had an effect on the genetic architecture of egg production and egg quality traits. As described in the Methods section, we estimated the interaction of all QTL detected with diet or age at egg collection. Forty-three QTL showed a significant interaction with diet, 12 QTL showed a significant interaction with age at egg collection and five QTL showed a significant interaction with both diet and age at egg collection, according to the Z test. A same QTL, i.e. the same location on the genome, could happen to be in interaction or not, depending on the trait. This is mostly due to the fact that the top SNP of a QTL can differ between traits. For example, QTL84 was detected for both ESshape and ESC but two different top SNPs were identified, i.e. AX-75934213, which showed an interaction with diet, and AX-80996918, which showed an interaction with age at egg collection.

#### Egg production (EPR, EPR1, EPR2, EPR3) traits

Among the 32 QTL detected for egg production traits, 26 had a significant interaction with diet (Table 5). Among the QTL that showed an interaction with diet, 21 explained a larger proportion of variance for the LE diet and five explained a larger proportion of variance for the HE diet. For EPR1, 22 QTL were detected of which 19 showed an interaction with diet. This suggests that EPR1 has a polygenic determinism and that it is highly subject to interaction with diet. For EPR2 and ERP3, only three and seven of the detected QTL, respectively, showed an interaction with diet. For these two traits, the QTL that showed an interaction with diet explained a larger proportion of variance for the LE diet than for HE diet. Finally, seven QTL were identified for EPR, of which QTL68 explained a larger proportion of variance for the HE diet.

**Table 5:** QTL with significantly different effects between diets

QTL	Trait	Chr	Var LE (%)	Var HE (%)	Z Diet
SNP5	EPR	0	5.06	1.16	4.41***
QTL7	EPR	2	5.44	2.61	2.75*
QTL23	EPR	4	4.8	0.54	9.27***
QTL47	EPR	9	4.92	0.53	7.14***
QTL68	EPR	11	0.02	4.41	7.78***
QTL79	EPR	17	2.39	0.83	14.23***
QTL90	EPR	20	4.89	0.01	8.48***
QTL7	EPR1	2	3.86	1.79	22.38***
QTL14	EPR1	3	5.98	2.1	20.68***
QTL28	EPR1	5	3.48	1.01	11.56***
QTL38	EPR1	7	2.64	1.12	18.8***
QTL39	EPR1	7	3.19	2.27	13.72***
QTL40	EPR1	7	3.01	1.37	13.76***
QTL42	EPR1	8	4.15	6.22	14.25***
QTL44	EPR1	9	1.42	3.57	3.74***
QTL46	EPR1	9	0.01	4.18	7.8***
QTL47	EPR1	9	5.52	0.46	7.28***
QTL51	EPR1	9	4.26	2.66	22.76***
QTL71	EPR1	12	0.02	4.26	7.04***
QTL75	EPR1	13	3.27	2.21	19.83***
QTL79	EPR1	17	5.85	1.68	21.41***
QTL90	EPR1	20	7.09	0.21	11.12***
QTL91	EPR1	21	4.2	1.05	12.59***
QTL99	EPR1	23	3.92	0.24	8.92***
QTL110	EPR1	27	3.48	1.19	14.93***
QTL116	EPR1	28	9.68	0	13.09***
QTL7	EPR2	2	4.9	1.84	3.66***
QTL67	EPR2	11	5.07	0.89	4.55***
QTL87	EPR2	20	4.46	0.68	6.46***
SNP4	EPR3	0	6.04	0.95	5.77***
SNP5	EPR3	0	6.87	0.9	6.39***
QTL7	EPR3	2	5.65	0.94	5.42***
QTL26	EPR3	4	3.19	0.07	15.03***
QTL51	EPR3	9	4.57	0.78	18.88***
QTL75	EPR3	13	3.6	1.3	18.47***
QTL90	EPR3	20	4.12	0.03	8.24***
QTL1	ESshape	1	1.88	5.4	3.58***
QTL45	ESshape	9	1.58	4.7	3.44***
QTL84	ESshape	19	1.29	4.11	5.9***
QTL59	EW	10	2.37	4.98	2.74*
QTL15	ESC	3	4.97	1.7	3.16**
QTL74	ESC	13	2.13	4.55	2.96**
QTL83	ESC	19	4.05	1.41	3.13**
QTL69	RSS	12	5.62	2.46	3.82***
QTL83	RSS	19	3.95	1.09	3.37**
QTL117	RSS	LGE22	0.47	2.41	4.97***
QTL13	YSS	3	4.88	2.12	2.87*
QTL15	YSS	3	5.84	1.61	3.98***
QTL16	YSS	3	4.96	1.72	3.45***
QTL52	YSS	9	4.34	0.64	4.43***
QTL56	YSS	10	4.33	0.9	7.3***
QTL82	YSS	19	4.26	0.26	5.93***
QTL11	ESS	2	1.81	5.71	3.84***
QTL102	ESS	24	4.89	1.3	3.01**
QTL117	ESS	LGE22	0.06	4.46	6.14***
QTL4	ESSTIF	1	7.56	2.94	3.45***
QTL85	ESSTIF	20	3.02	1.19	4.38***
QTL46	HU	9	1.22	5.17	3.95***
QTL110	HU	27	1.73	4.49	3.55***
QTL19	EMTSP	3	2.75	4.92	2.77*
QTL87	EMTSP	20	1.24	3.37	4.04***
QTL100	EMTSP	24	1.46	3.91	2.84*
QTL62	YOLKIND	10	5.14	0.89	3.03**
QTL105	YOLKIND	26	4.19	0.64	3.55***

*Chr* chromosome that carries the QTL, *var LE* variance explained for the low energy diet, *var HE* variance explained for the high energy diet, *LGE22* linkage group LGE22C19W28_E50C23, *Z Diet* Z test statistics for QTL × diet interaction

* P < 0.1, ** P < 0.05, *** P < 0.01

#### Egg weight (EW) and egg form (SLE and ESshape) traits

Among the 27 QTL previously detected for egg weight and egg form, four showed an interaction with diet and one showed a significant interaction with age. All the QTL that showed an interaction with diet accounted for a larger proportion of variance explained for the HE diet than for the LE diet. QTL59 was detected for EW on chromosome 10 and had a greater effect for the HE diet than for the LE diet (5.0 % of variance explained *vs.* 2.4 %). The three other QTL which had an interaction with diet, QTL1, QTL45 and QTL84 were detected for ESshape. QTL101, which showed an interaction with age at egg collection, explained a larger proportion of variance for ESshape for egg collection at 50 weeks of age (4.2 *vs.* 1.3 %) than at 70 weeks of age. None of the QTL detected for SLE showed an interaction with age at egg collection or diet. Thus, this trait does not seem to be sensitive to environment, or at least it was metabolically stable regardless of age at egg collection or diet.

#### Egg shell color (ESC, RSS, LSS and YSS) traits

Among the 39 QTL detected for egg shell color, 10 showed an interaction with diet, one an interaction with age at egg collection and one an interaction with both age at egg collection and diet (see Additional files 1, 2, 3). The QTL that showed an interaction with diet were located on chromosomes 3, 9, 10, 12, 13 and 19, and nine of these 10 QTL had a greater effect for the LE diet than for the HE diet (Table 5). QTL117, which was located on LGE22C19W28_E50C23 and had AX-80867378 as the top SNP, showed an interaction with both diet and age at egg collection. It accounted for a larger proportion of variance for egg collection at 70 weeks of age and for the HE diet (Table 5). QTL84 was identified on chromosome 19 around 5 Mb and its effect varied with age at egg collection. For LSS, no QTL showed an interaction with either age at egg collection or diet, which suggests that it is not sensitive to variations in environment.

#### Egg shell solidity (ESSTIF and ESS) traits

Among the 15 QTL detected for egg shell solidity, four showed an interaction with diet, three with age at egg collection and one with both diet and age at egg collection (Table 5). Among the QTL that showed an interaction with diet, QTL4 for ESSTIF and QTL102 for ESS accounted for a larger proportion of variance for the LE diet than for the HE diet, and QTL11 and QTL117 for ESS accounted for a larger proportion of variance for the HE diet than for the LE diet. The three QTL that showed an interaction with age at egg collection (QTL18, QTL72 and QTL103) were related to ESS and accounted for a larger proportion of variance for egg collection at 70 weeks of age. QTL85, detected for ESSTIF, showed an interaction with both diet and age and accounted for a larger proportion of variance for the LE diet and for egg collection at 50 weeks of age. The same number of QTL was detected for ESSTIF and ESS but, for ESS, more QTL showed an interaction with diet or age, which suggests that ESS is more sensitive to environment than ESSTIF.

#### Internal egg quality (YOLKIND, HU and EMTSP) traits

Among the 33 QTL detected for internal egg quality traits, five showed an interaction with diet, nine with age at egg collection and two with both diet and age at egg collection. Among the QTL that showed an interaction with diet only, three (QTL46, QTL87 and QTL110) accounted for a larger proportion of variance for the HE diet than for the LE diet. The two other QTL (QTL62 and QTL105), which were detected for YOLKIND, accounted for a larger proportion of variance for the LE diet than for the HE diet. Among the QTL that showed an interaction with age, four (QTL21, QTL25, QTL111 and QTL113) accounted for a larger proportion of variance for egg collection at 50 weeks of age. The five other QTL (QTL17, QTL33, QTL78, QTL93 and QTL100) accounted for a larger proportion of variance for egg collection at 70 weeks of age. The two QTL that showed an interaction with both diet and age were detected for EMTSP and accounted for a larger proportion of variance for the HE diet and for egg collection at 50 weeks of age.

## Conclusions

This study detected 131 QTL for egg production and egg quality traits. These QTL were distributed across 27 chromosomes, two linkage groups (LGE22C19W28_E50C23 and LGE64) and a group of unassigned SNPs (Table 6). Among the 131 QTL detected, 60 showed a significant interaction with age at egg collection and/or diet (Table 5) although the average phenotypic performance varied only slightly with diet and age at collection (Table 1). This shows that laying hens have an in-built ability to adapt to their environment that probably involves different genetic pathways. These complex G × E interactions could have an effect on genetic selection, since the best candidates may differ depending on the environmental conditions in which the hens are reared. Performance of crossbred daughters was used to characterize pure line sires. This study pinpoints the existence of “unrobust” QTL, which raises the question which QTL are expressed in the commercial hybrids. To answer this, QTL detection based on the genotypes of commercial hybrids would be useful.

**Table 6:** Number and names of QTL detected on each chromosome and traits affected

Chr	Nb QTL	QTL	Trait
0	5	SNP5, SNP4, SNP2, SNP1, SNP3	EPR, EPR3, ESC, ESshape, EW, LSS, RSS
1	5	QTL1, QTL2, QTL4, QTL3, QTL5	ESshape, ESSTIF, YOLKIND
2	7	QTL7, QTL9, QTL10, QTL11, QTL8, QTL12, QTL6	EPR, EPR1, EPR2, EPR3, ESC, ESS, ESSTIF, LSS, RSS, YSS
3	10	QTL19, QTL14, QTL15, QTL20, QTL18, QTL17, QTL21, QTL13, QTL16, QTL22	EMTSP, EPR1, ESC, ESS, HU, LSS, RSS, YSS
4	4	QTL23, QTL26, QTL24, QTL25	EPR, EPR3, EW, HU
5	6	QTL28, QTL30, QTL31, QTL27, QTL32, QTL29	EPR1, ESC, ESshape, LSS, RSS
6	4	QTL35, QTL33, QTL34, QTL36	EW, HU, YOLKIND
7	4	QTL38, QTL39, QTL40, QTL37	EPR1, ESSTIF
8	3	QTL43, QTL42, QTL41	EMTSP, EPR1, ESSTIF
9	11	QTL44, QTL47, QTL46, QTL51, QTL48, QTL49, QTL53, QTL54, QTL50, QTL45, QTL52	EPR, EPR1, EPR2, EPR3, ESC, ESS, ESshape, HU, LSS, RSS, YSS
10	10	QTL60, QTL61, QTL58, QTL60-61, QTL59, QTL63, QTL55, QTL62, QTL56, QTL57	ESC, ESshape, EW, LSS, YOLKIND, YSS
11	5	QTL65, QTL68, QTL67, QTL66, QTL64	EPR, EPR2, EW, RSS, SLE
12	5	QTL70, QTL71, QTL73, QTL72, QTL69	EMTSP, EPR1, ESC, ESS, LSS, RSS
13	2	QTL75, QTL74	EPR1, EPR3, ESC
14	1	QTL76	EPR1
15	2	QTL77, QTL78	HU, YOLKIND
17	2	QTL79, QTL80	EPR, EPR1, ESC, LSS, RSS
18	1	QTL81	ESC, LSS, YSS
19	3	QTL82, QTL83, QTL84	ESC, ESshape, LSS, RSS, YSS
20	6	QTL87, QTL90, QTL85, QTL86, QTL88, QTL89	EMTSP, EPR, EPR1, EPR2, EPR3, ESSTIF, YOLKIND
21	4	QTL91, QTL94, QTL93, QTL92	EPR1, EW, HU, SLE, YOLKIND
22	2	QTL96, QTL95	ESSTIF, LSS
23	3	QTL99, QTL97, QTL98	EPR1, ESC, ESshape, LSS
24	5	QTL100, QTL102, QTL103, QTL101, QTL104	EMTSP, ESS, ESshape, ESSTIF, HU, SLE
26	5	QTL106, QTL109, QTL108, QTL107, QTL105	ESshape, EW, HU, SLE, YOLKIND
27	2	QTL110, QTL111	EPR1, HU
28	6	QTL115, QTL116, QTL114, QTL113-114, QTL112, QTL113	EPR1, EPR2, ESS, YOLKIND
LGE22	1	QTL117	ESS, ESshape, RSS, YOLKIND
LGE64	1	QTL118	ESshape, YSS
Z	8	QTL120, QTL123, QTL124, QTL125, QTL119, QTL122, QTL126, QTL121	EMTSP, ESC, ESshape, EW, LSS, RSS, SLE, YSS

*Chr* chromosome that carries the QTL, *Nb QTL* number of QTL detected on each chromosome

## Additional files

10.1186/s12711-015-0160-2 QTL detected using the whole dataset to determine the genetic architecture of egg production and egg quality traits. This file gives the position of all the QTL detected using the whole dataset, with the top SNP corresponding to the SNP with the highest effect in the QTL region. The QTL is defined by the first (left) and last (right) SNPs that are 1 % significant at the chromosome level, respectively. Var (%) is the percentage of variance explained by the top SNP in the analysis with the whole dataset. Var LE(%) is the percentage of variance explained by the top SNP in the analysis with data for the low-energy diet only. Var HE(%) is the percentage of variance explained by the top SNP in the analysis with data for the high-energy diet only. Var 50(%) is the percentage of variance explained by the top SNP in the analysis with data for egg collection at 50 weeks only. Var 70(%) is the percentage of variance explained by the top SNP in the analysis with data for egg collection at 70 weeks only. Z Diet is the Z test statistics used to compare the two estimates calculated from the data for LE and HE diets. Z Age is the Z test statistics used to compare the two estimates calculated from the data for egg collection at 50 and 70 weeks of age. The difference was significant when P < 0.1 *, P < 0.05 ** and P < 0.01***.
10.1186/s12711-015-0160-2 Additional QTL detected using the data for egg production and egg quality traits within diet. This file gives the position of the QTL detected with the dataset divided by diet, with the top SNP corresponding to the SNP with the highest effect in the QTL region. The QTL is defined by the first (left) and last (right) SNPs that are 1 % significant at the chromosome level, respectively. Var (%) is the percentage of variance explained by the top SNP in the analysis with the whole dataset. Var LE(%) is the percentage of variance explained by the top SNP in the analysis with data for the low-energy diet only. Var HE(%) is the percentage of variance explained by the top SNP in the analysis with data for the high-energy diet only. Z Diet is the Z test statistic used to compare the two estimates calculated from the data for LE and HE diets. The difference was significant when P < 0.1 *, P < 0.05 ** and P < 0.01***.
10.1186/s12711-015-0160-2 Additional QTL detected using the data for egg quality traits according to age. This file gives the position of the QTL detected with the dataset divided by age, with the top SNP corresponding to the SNP with the highest effect in the QTL region. The QTL is defined by the first (left) and last (right) SNPs that are 1 % significant at the chromosome level, respectively. Var (%) is the percentage of variance explained by the top SNP in the analysis with the whole dataset. Var 50(%) is the percentage of variance explained by the top SNP in the analysis with data for egg collection at 50 weeks only. Var 70(%) is the percentage of variance explained by the top SNP in the analysis with data for egg collection at 70 weeks only. Z Age is the Z test statistic used to compare the two estimates calculated from the data for egg collection at 50 and 70 weeks of age. The difference was significant when P < 0.1 *, P < 0.05 ** and P < 0.01***.

## References

[1] Chicken QTL database. Iowa State University 2003. [http://www.animalgenome.org/cgi-bin/QTLdb/GG/index]. Accessed 13 Mar 2015.

[2] Kranis A, Gheyas AA, Boschiero C, Turner F, Yu L, Smith S (2013). Development of a high density 600K SNP genotyping array for chicken. BMC Genomics.

[3] El-Soda M, Malosetti M, Zwaan BJ, Koornneef M, Aarts MGM (2014). Genotype × environment interaction QTL mapping in plants: lessons from Arabidopsis. Trends Plant Sci.

[4] Atol Ontology. INRA 2012. [http://www.atol-ontology.com]. Accessed 11 Mar 2015.

[5] Eisen EJ, Bohren BB, McKean HE (1962). The Haugh unit as a measure of egg albumen quality. Poult Sci.

[6] Teyssèdre S, Elsen JM, Ricard A (2012). Statistical distributions of test statistics used for quantitative trait association mapping in structured populations. Genet Sel Evol.

[7] Falconer DS, Mackay TFC (2009). Introduction to quantitative genetics.

[8] Misztal I, Tsuruta S, Strabel T, Auvery B, Druet T, Lee DH. BLUPF90 and related programs (BGF90). In Proceedings of the 7th World Congress on Genetics Applied to Livestock Production. Montpellier; 2002. p. 19–23.

[9] Müller BU, Stich B, Piepho H-P (2011). A general method for controlling the genome-wide type I error rate in linkage and association mapping experiments in plants. Heredity (Edinb).

[10] Ricard A, Filangi O, Elsen JM. GWAS Muller—Guide de l’utilisateur. Version 3.0.0. 2013. Accessed 29 Sept 2015.

[11] Atzmon G, Blum S, Feldman M, Lavi U, Hillel J (2007). Detection of agriculturally important QTLs in chickens and analysis of the factors affecting genotyping strategy. Cytogenet Genome Res.

[12] Tuiskula-Haavisto M, de Koning DJ, Honkatukia M, Schulman NF, Mäki-Tanila A, Vilkki J (2004). Quantitative trait loci with parent-of-origin effects in chicken. Genet Res.

[13] Liu W, Li D, Liu J, Chen S, Qu L, Zheng J (2011). A genome-wide SNP scan reveals novel loci for egg production and quality traits in white leghorn and brown-egg dwarf layers. PLoS One.

[14] Tuiskula-Haavisto M, Honkatukia M, Vilkki J, de Koning DJ, Schulman NF, Mäki-Tanila A (2002). Mapping of quantitative trait loci affecting quality and production traits in egg layers. Poult Sci.

[15] Wolc A, Arango J, Settar P, Fulton JE, O’Sullivan NP, Preisinger R (2012). Genome-wide association analysis and genetic architecture of egg weight and egg uniformity in layer chickens. Anim Genet.

[16] Schreiweis MA, Hester PY, Settar P, Moody DE (2006). Identification of quantitative trait loci associated with egg quality, egg production, and body weight in an F2 resource population of chickens. Anim Genet.

[17] Wolc A, Arango J, Jankowski T, Dunn I, Settar P, Fulton JE (2014). Genome-wide association study for egg production and quality in layer chickens. J Anim Breed Genet.

[18] Honkatukia M, Tuiskula-Haavisto M, Ahola V, Uimari P, Schmutz M, Preisinger RD (2011). Mapping of QTL affecting incidence of blood and meat inclusions in egg layers. BMC Genet.

